# A portable luminescent thermometer based on green up-conversion emission of Er^3+^/Yb^3+^ co-doped tellurite glass

**DOI:** 10.1038/srep41596

**Published:** 2017-01-31

**Authors:** Danilo Manzani, João Flávio da Silveira Petruci, Karina Nigoghossian, Arnaldo Alves Cardoso, Sidney J. L. Ribeiro

**Affiliations:** 1 Institute of Chemistry, São Paulo State University, UNESP, CP 355, Araraquara, SP, Brazil

## Abstract

The determination of temperature is essential in many applications in the biomedical, technological, and industrial fields. Optical thermometry appears to be an excellent alternative for conventional electric temperature sensors because it is a non-contact method that offers a fast response, electromagnetic passivity, and high temperature sensitivity. In this paper, we propose an optical thermometer probe comprising an Er^3+^/Yb^3+^ co-doped tellurite glass attached to the tip of an optical fibre and optically coupled to a laser source and a portable USB spectrometer. The ratio of the up-conversion green emission integrated peak areas when excited at 980 nm was temperature dependent, and it was used to calibrate the thermometer. The thermometer was operated in the range of 5–50 °C and 50–200 °C, and it revealed excellent linearity (*r*^2^ > 0.99), suitable accuracy, and precisions of ±0.5 and ±1.1 °C, respectively. By optimizing Er^3+^ concentration, we could obtain the high green emission intensity, and in turn, high thermal sensitivity for the probe. The probe fabricated in the study exhibited suitable properties for its application as a temperature sensor and superior performance compared to other Er^3+^ -based optical thermometers in terms of thermal sensitivity.

Temperature is a fundamental parameter as it governs various chemical and physical intracellular interactions that occur in the life cycle of biological cells^1^,^2^,^3^. In the biomedical field, there is an increasing demand for temperature sensors that can measure temperature at the milli- and micro-scale in order to diagnose pathologies and optimise therapeutic processes^4^,^5^. Additionally, the determination of temperature is essential in many applications, including military, industrial, environmental, and technological applications. Conventional contact sensors based on electrical changes in the material caused by temperature variations have limitations such as a slow response and high sensitivity to electromagnetic interference. Such sensors are not suitable in a variety of applications, such as for measuring the electrical transformer temperature in power stations or the temperature in oil refineries, pipelines, coal mines, and building fires^6^,^7^. Furthermore, the development of inexpensive, reliable, portable, and safe temperature sensors for an extended range of applications is essential.

Within the last few decades, research in optical thermometry has increased because of its advantages, such as its fast response, electromagnetic passivity, and high temperature sensitivity^6^,^8^. Sensors based on the temperature-dependent fluorescence intensity ratio (FIR) of active ion-containing hosts in different materials, such as glass ceramics^7^, nanoparticles^9^ and glasses^10^, have been increasingly reported. Additionally, in terms of sensor instrumentation, optically based devices present several advantages, such as the fact that there is no need to convert electronics to photonics or vice versa, which reduces their cost and increases their flexibility for different applications. In addition, the sensing element (an optically active doped material) must be highly stable across a broad range of temperatures, present efficient luminescence, and be resistant against chemical vapours and humidity^8^,^11^.

Various optical thermometry techniques have been studied in the past few decades via the luminescence of probes such as metal–ligand complexes^5^,^12^, phosphor thermometry^13^,^14^, and quantum dots^15^,^16^. The FIR technique is a versatile method and is widely used to determine temperature based on measurement of the fluorescence intensities from two thermally coupled energy levels in rare-earth ions (RE^3+^) because it improves the accuracy, resolution, and reliability. It also reduces the impact of the experimental conditions, such as fluorescence loss and fluctuation of the pumping power^6^,^17^,^18^,^19^. Among RE^3+^, praseodymium^20^, neodymium^21^, europium^22^, holmium^21^, and erbium^18^,^23^,^24^ have been used for optical temperature sensing because they generate emissions that are dependent on temperature owing to the presence of thermally coupled levels. The population of these levels follows a Boltzmann-type population distribution at quasi-thermal equilibrium. The difference of energy between these levels must be larger than ~200 cm^−1^ to avoid strong overlapping of the two emitted wavelengths and it should be shorter than 2000 cm^−1^ to allow the upper level to have a minimum population of RE^3+^ in the temperature range of interest^6^,^8^.

Erbium is the most studied active ion in glass for optical thermometry because it exhibits up-conversion (UC) fluorescence emissions upon excitation using infrared (IR) radiation. However, neodymium is also gaining attention as a sensitizer in fluorescent bioprobes for excitation at 800 nm rather than at 980 nm. Because water absorption at 800 nm is significantly lower than that at 980 nm, the activation of UC fluorescence at such wavelengths allows for minimal heating of tissues^25^. Differently doped glass matrixes have been studied for this purpose, such as chalcogenide^26^, fluoroindate^18^, fluorophosphates^24^, tellurite^27^, and fluorotellurite^28^ glass. Erbium ions have a thermally coupled pair of energy levels, ^2^H_11/2_ and ^4^S_3/2_, whose green emission intensity ratio varies with temperature, allowing it to act as a probe for measuring environmental temperature where it is inserted^29^. However, the relatively low absorption cross section of Er^3+^ of approximately 6 × 10^−21^ cm^2^ at 980 nm^30^ results in weak visible emission efficiencies. Co-doping Yb^3+^ in Er^3+^ -doped materials significantly improves the visible UC emission intensities by increasing the absorption cross section to approximately 12 × 10^−21^ cm^2^. Yb^3+^ absorbs the 980 nm light more efficiently and then transfers the energy to Er^3+^ owing to the large spectral overlap between Yb^3+^ emission (^2^F_5/2_ → ^2^F_7/2_) and Er^3+^ absorption (^4^I_11/2_ ← ^4^I_15/2_). For practical application, the use of an IR excitation source instead of an ultraviolet (UV) excitation source has the advantage of being commercially less expensive and more portable, in addition to being more suitable for biological applications.

Optical temperature sensors based on luminescent ions must have high radiative probabilities for the emitting levels to obtain high emission intensities, which are directly related to the glass matrix in which the RE^3+^ are embedded. Among the various types of oxide glass, tellurite glass (TG) presents interesting properties that drive their use as a host material for many different RE^3+^. Tellurite-based glass presents a high capacity for RE^3+^ loading without clustering, phase separation, or crystallisation of the doping species. In addition to its large transparency in the visible region from 300 to 800 nm, tellurite has a relatively low phonon energy (750 cm^−1^), which makes it suitable as an RE^3+^ host as it leads to reduced multiphonon relaxation of the ion excited states, which has a strong impact on the energy transfer process. Specifically for temperature sensing, the glass transition temperature (*T*_g_) must be taken in account because it is a thermal event that marks the smooth passage of a glass to the super-cooled molten state while heating, causing changes in several properties such as volume, viscosity, and heat capacity^31^. In this sense, the glass transition region is the limiting parameter for the temperature range in which the glass temperature sensor can be applied. Tellurite-based glass is commonly called “soft glass” because of its low characteristic temperature; its *T*_g_ is approximately 250 to 350 °C, depending on the composition. For biological systems, the clinically relevant range for monitoring temperature is up to 60 °C; biological molecules are subject to denaturing above this temperature^4^.

In this contribution, we report the development of a low-cost and portable solution for determining temperature. The FIR of the UC green emission in Er^3+^/Yb^3+^ co-doped tellurite glass is temperature dependent, and thus it was used for quantification based-calibration of the optical thermometer. Herein, we have followed a systematic strategy to calibrate and evaluate the thermometer performance in terms of its accuracy, precision, and calibration parameters. Moreover, a simple, low-cost, portable, and easy-to-use instrumentation setup was employed in the optical system.

## Results and Discussion

### Glass samples and thermal analysis

**Figure 1:**
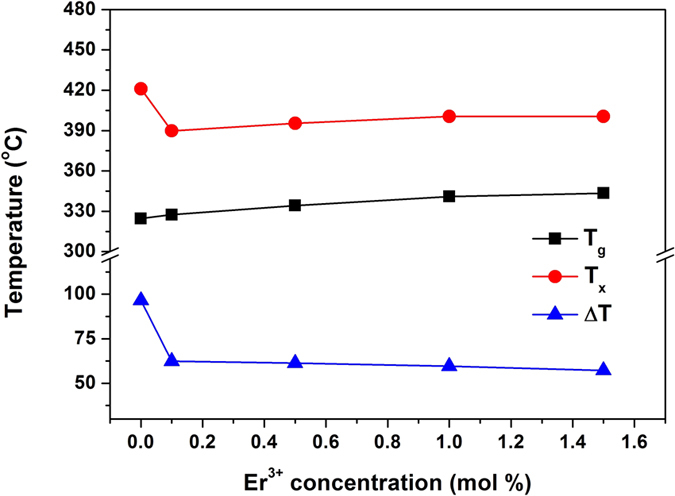
Variation of the characteristic temperatures, *T*_*g*_, *T*_*x*_, and Δ*T*, of the undoped and Er^3+^/Yb^3+^ co-doped (97−*x*)[70TeO_2_–15GeO_2_–5K_2_O–10Bi_2_O_3_]:*x*Er_2_O_3_/_3_Yb_2_O_3_ glass samples as a function of Er^3+^ concentration.

Colourless non-doped and slightly pink co-doped glass samples were obtained from glass compositions of (97−*x*)[70TeO_2_–15GeO_2_–5K_2_O–10Bi_2_O_3_]:*x*Er_2_O_3_/3Yb_2_O_3_ (mol%), where *x* = 0.1, 0.5, 1.0, and 1.5 mol%. From the DSC curves recorded for the TG samples, we determined the glass transition temperature, *T*_*g*_ (±2 °C), the onset temperature of crystallisation, *T*_*x*_ (±2 °C), and the glass thermal stability against crystallisation, Δ*T* = *T*_*x*_−*T*_*g*_ (±3 °C). The variations of the characteristic temperatures are shown in Fig. 1. First, small increases of *T*_*g*_ among the co-doped samples from 327 °C to 343 °C and of *T*_*x*_ from 390 °C to 401 °C show that Er^3+^ was well incorporated into the glass network. However, the slight decrease of Δ*T* (from 63 °C to 57 °C) reveals that the addition of Er^3+^ began to alter the glass network connectivity. From the point of view of temperature-sensing applications, the maximum temperature at which these glass compositions can be safely applied without compromising any structural, thermal, or thermomechanical properties is 30 to 50 °C below *T*_*g*_.

### Absorption and emission spectra

**Figure 2:**
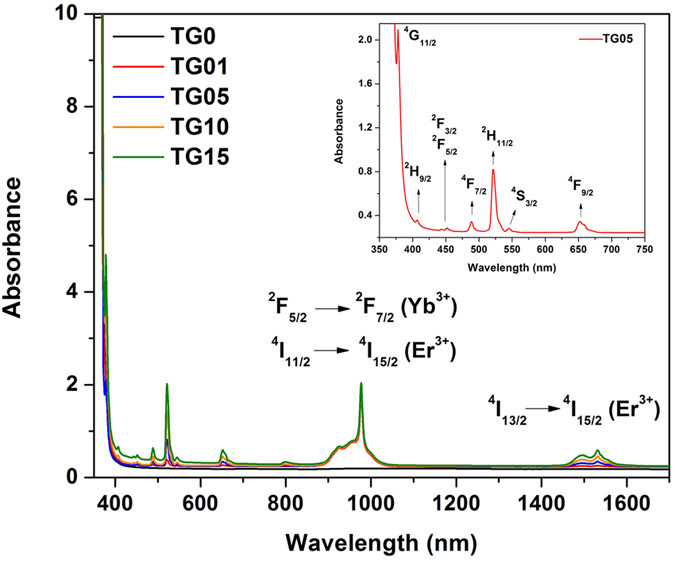
UV–vis–NIR absorption spectra of the undoped (TG0) and co-doped samples (TG01, TG05, TG10, TG15). Inset shows the magnification of the visible region with Er^3+^ excited levels assigned, considering their absorption from their ground state, ^4^I_15/2_.

TG glasses containing different Er^3+^ and Yb^3+^ concentrations (except the TG01 sample) were pinkish in colour and homogeneous in appearance. Figure 2 depicts the absorption spectra of these samples from 350 to 1700 nm. Characteristic absorption bands of Er^3+^ are located at 1432, 976, 652, 545, 521, 488, 450, 443, 407, and 378 nm, which were ascribed to transitions from the ground state, ^4^I_15/2_, to the excited states, ^4^I_13/2_, ^4^I_11/2_, ^4^F_9/2_, ^4^S_3/2_, ^2^H_11/2_, ^4^F_7/2_, ^2^F_5/2_, ^2^F_3/2_, ^2^H_9/2_, and ^4^G_11/2_, respectively. The inset of Fig. 2 shows the magnification of the visible region containing Er^3+^ absorption bands. For Yb^3+^ ions, the absorption band is located from 890 to 1020 nm, which overlaps with the Er^3+^ absorption bands and is attributed to the transition from the ground state, ^2^F_7/2_, to the excited state, ^2^F_5/2_. As expected, no change was observed in the position or intensity of the Yb^3+^ absorption band. The only change was the enhanced intensity of the Er^3+^ absorption bands owing to the increased Er_2_O_3_ content.

**Figure 3:**
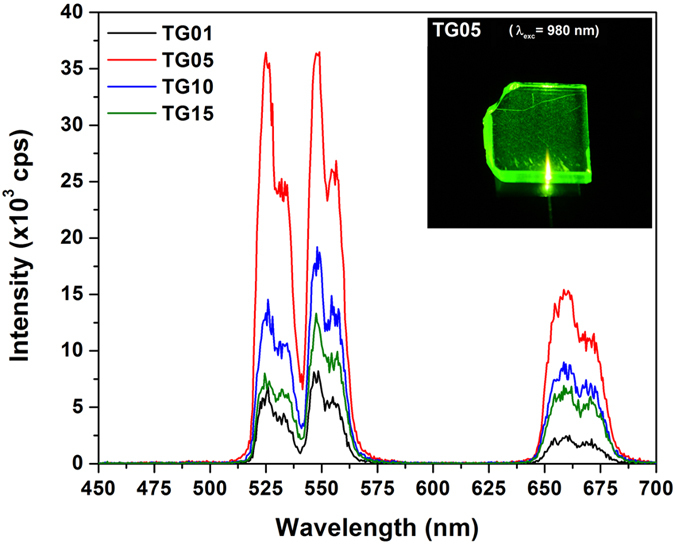
Visible UC emission spectra of Er^3+^/Yb^3+^ co-doped in the TG samples under excitation at 980 nm with 150 mW laser power as a function of Er3+ concentration. Inset shows the bright-green emission of the TG05 sample.

**Figure 4:**
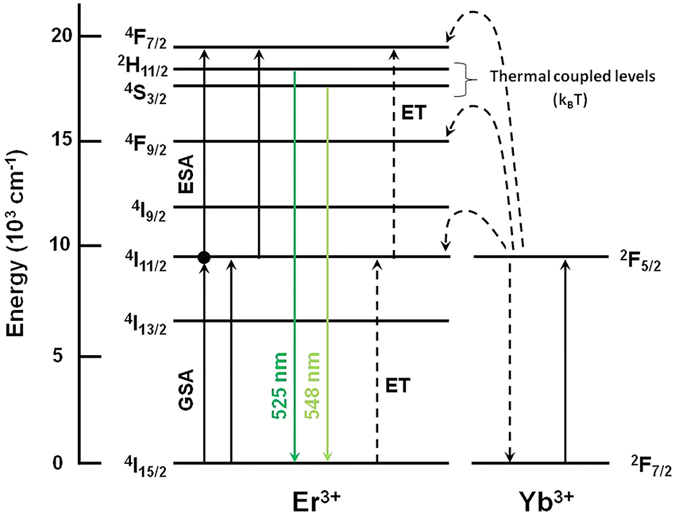
The energy-level diagram of the green UC emissions for the Er^3+^/Yb^3+^ co-doped TG glass under 980 nm laser excitation.

The visible UC emission spectra were recorded in the 400–700 nm range for the TG01, TG05, TG10, and TG15 samples upon 980 nm diode laser excitation with 150 mW, and the results are shown in Fig. 3. The inset of this figure shows a photograph of the TG05 glass emitting a bright-green light visible by the naked eye. With increasing Er_2_O_3_ content from 0.1 to 0.5 mol%, the intensity of the visible emission in both the green and red regions gradually increased; it then decreased for concentrations from 0.5 to 1.5 mol%. This fact can be accounted for by the decreasing distance between Er^3+^ and Yb^3+^, which enhanced the efficiency of energy level interactions between them before attaining the optimum co-doping concentrations of 0.5 mol% Er_2_O_3_ and 3 mol% Yb_2_O_3_. The decrease of visible luminescence intensity above 0.5 mol% Er_2_O_3_ is explained by the drastic reduction of the distance between neighbouring Er^3+^ atoms, which increased the Er–Er interaction. This increased interaction led to the quenching of the green UC emissions and the enhanced energy transfer rate from erbium to the OH^−^ groups^10^,^31^. At high concentrations of Er^3+^ and with significant overlapping of the emission and absorption green bands, radiative transfer by re-absorption is favoured, thus reducing sensitivity^10^. For optical temperature sensing, the concentration of Er^3+^ (activator ion) is an important factor that must be evaluated. Wang *et al*. showed that a material with low Er^3+^ doping had approximately twice the optical temperature sensitivity of a highly doped one^32^. In this study, by using a high concentration of Yb^3+^ (sensitizer) co-doped with relatively low concentrations of Er^3+^ (activator, up to 1.5 mol%) in a tellurite glass, it was possible to obtain very intense green emission and thermal sensitivity, thus avoiding re-absorption. These ions are excited because of the addition of photons by the transfer energy effect (APTE, from “Addition de Photons par Transferts d’Energie”) from the Yb^3+^ ions, which act as sensitizers^33^,^34^. The APTE effect is 10^5^ times more efficient than the cooperative luminescence process that is typical in Yb^3+^ -doped samples at high concentrations for the same Yb^3+^ –Yb^3+^ distances^35^,^36^,^37^. This is because of the larger absorption cross section of Yb^3+^ for 980 nm radiation, and it easily transfers its absorbed energy to Er^3+^ through the ^2^F_5/2_ (Yb^3+^) → ^4^I_11/2_ (Er^3+^) levels, thus enhancing the population of the ^4^I_11/2_ level of Er^3+^ many times (Fig. 4).

Although all samples exhibited UC emissions, the TG05 sample showed the highest intensity. The maximum intensity emissions at 525 and 548 nm originating from Er^3+^ are assigned to the ^2^H_11/2_ → ^4^I_15/2_ and ^4^S_3/2_ → ^4^I_15/2_ transitions, respectively. For the red emission, a broad band centred at 660 nm is attributed to the ^4^F_9/2_ → ^4^I_15/2_ transition of Er^3+^. For temperature sensor applications, the green emissions are responsible for conveying the temperature information from the medium in which the sensor is inserted. In Er^3+^/Yb^3+^ co-doped TG glass, three possible UC mechanisms mainly occur for green emissions: excited state absorption (ESA), energy transfer (ET)^36^, and cross relaxation (CR)^32^. Figure 4 shows the energy-level diagram of Er^3+^ and Yb^3+^ and the mechanisms involved. For green emissions, a two-step UC mechanism may occur: (i) the excited-state ^4^I_11/2_ level of Er^3+^ is populated by absorption of the 980 nm pump light from the ground state, ^4^I_15/2_ (GSA), and by energy transfer (ET) from the excited state of Yb^3+^, ^2^F_5/2_, owing to the absorption of the 980 nm light by the ground-state ^2^F_7/2_ level: ^2^F_5/2_ (Yb^3+^) + ^4^I_15/2_ (Er^3+^) → ^2^F_7/2_ (Yb^3+^) + ^4^I_11/2_ (Er^3+^); (ii) the excitation processes involving electrons at the ^4^I_11/2_ (Er^3+^) excited state by different mechanisms: (a) by ESA, where an excited Er^3+^ ion absorbs a second 980 nm photon, raising the electrons to the highest energy level through ^4^I_11/2_ (Er^3+^) + photon → ^4^F_7/2_ (Er^3+^), and (b) by energy transfer (ET) of ^2^F_5/2_ (Yb^3+^) + ^4^I_11/2_ (Er^3+^) → ^2^F_7/2_ (Yb^3+^) + ^4^F_7/2_ (Er^3+^). Moreover, a CR mechanism may be considered in excited Yb^3+^ (^2^F_7/2_ + 980 nm photon → ^2^F_5/2_) and Er^3+^ ions of ^4^I_11/2_ + ^4^I_11/2_ → ^4^I_15/2_ + ^4^F_7/2_^38^. Then, non-radiative decay of the excited electrons in the ^4^F_7/2_ level take place, relaxing through matrix multiphonon processes to the lower energy levels of ^2^H_11/2_ and ^4^S_3/2_, which produce the final green UC emissions of Er^3+^ by the transitions of ^2^H_11/2_ → ^4^I_15/2_ (525 nm) and ^4^S_3/2_ → ^4^I_15/2_ (548 nm). These green emitter levels are separated by a very close energy gap of Δ*E* = 799.4 cm^−1^, and they are responsible for the emissions at 525 and 548 nm at room temperature, which allows the ^2^H_11/2_ level be thermally populated by the ^4^S_3/2_ level, which has been observed previously in other studies^11^,^18^,^23^,^27^,^28^,^29^.

**Figure 5:**
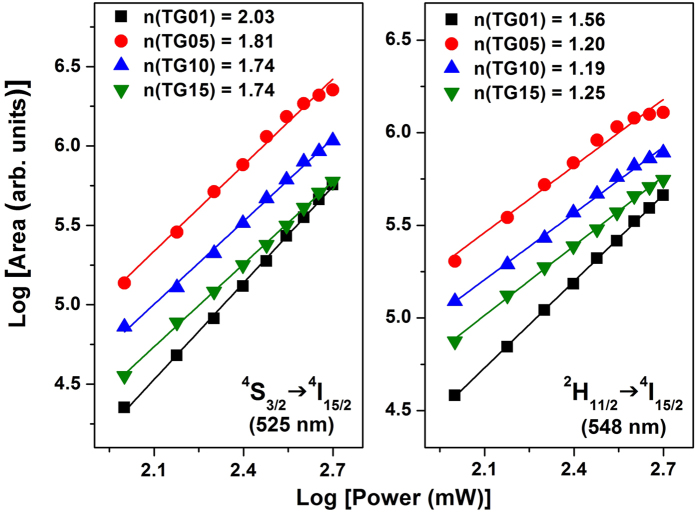
Green UC emission intensities as a function of excitation power operating at 980 nm for the TG01, TG05, TG10, and TG15 glass samples.

To better comprehend the UC mechanisms involved for the green emissions, the pump power dependence of the integrated band intensities for each green emission is shown in Fig. 5. In UC processes, the emission intensity, *I*_*UC*_, is proportional to *I*^*n*^_*ex*_, where *I*_*ex*_ is the intensity of the 980 nm excitation and *n* is the number of IR photons absorbed per visible photon emitted^39^, which is determined from the slope of the linear fit of log(*I*_*UC*_) vs. log(*I*_*ex*_). Figure 5 shows the logarithmic plots of *I*_*UC*_ vs. *I*_*ex*_ for the two green emissions, with good linear adjustment with the experimental data for all TG-doped samples (*R* > 0.99). The numbers of photons involved in green emissions at 525 and 548 nm for TG01, TG05, TG10, and TG15 were 2.03, 1.81, 1.74, and 1.74 and 1.56, 1.20, 1.19, and 1.25, respectively. The proposed UC mechanism indicates an average of two photons involved in the frequency UC process. However, contrary to the expectation that two photons were involved, the lower values obtained indicate the influence of temperature coming from the relatively high power of the excitation light focused on the glass sample surfaces. The relative populations in the emitting Er^3+^ levels, ^2^H_11/2_ and ^4^S_3/2_, are temperature dependent according to *k*_*B*_*T*, where *k*_*B*_ is the Boltzmann constant and *T* is the absolute temperature, which affects the intensity ratio between the two green emissions at 525 and 548 nm.

**Figure 6:**
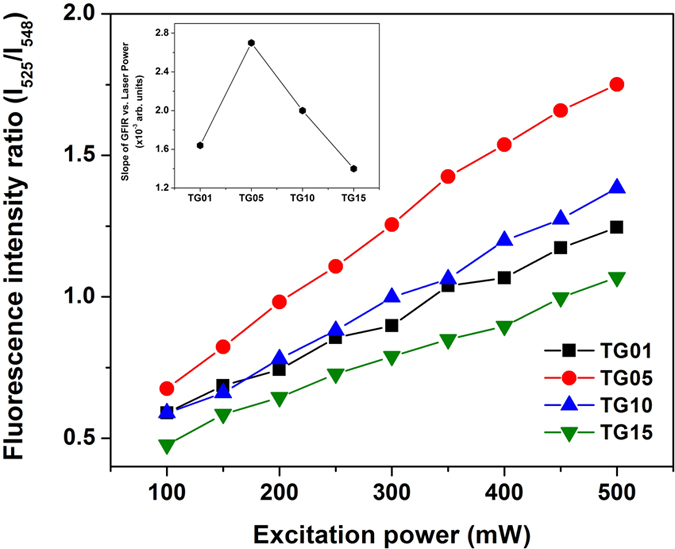
Variation of fluorescence intensity ratio of 525 and 548 nm green emissions as a function of 980 nm laser power varying from 100 to 500 mW. The laser beam was focused on the glass sample surfaces using a 13-mm-focal length lens.

The local increase in the temperature of the glass samples induced by the excitation light when the laser pump power varied from 100 to 500 mW at 980 nm is reflected in the slope of the FIR (I_525_/I_548_) vs. excitation power (mW) curves, shown in Fig. 6. This is the first evidence that these types of glass are sensitive to temperature changes, and thus they could potentially be applied as sensor materials in optical thermometry analysis. The inset of Fig. 6 shows the slopes of these curves as a function of Er^3+^ concentration; these curves are similar to the green UC intensity variations shown in Fig. 3. The steepest inclination in the yield curve was found for the TG05 sample, and it had the highest intensity green emission. Therefore, this sample was the one that best responded to temperature variations among the samples studied.

**Figure 7:**
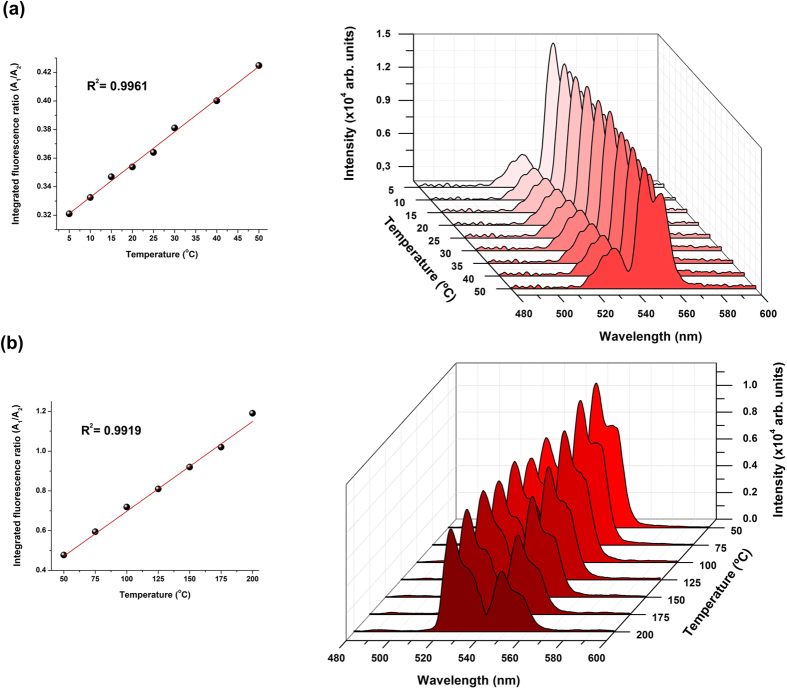
Temperature dependence of the Er^3+^ green up-converted emission for the co-doped tellurite glass doped with 0.5 mol% Er^3+^ and 3 mol% Yb^3+^ for (**a**) low temperature (LT) and (**b**) high temperature (HT) ranges. All the spectra were normalised to the maximum intensity of each spectrum.

Based on the aforementioned discussion, in order to obtain high sensitivity to temperature variations, the TG05 glass sample was chosen as the sensor material for application in the proposed portable optical thermometer operating at low temperatures from 5 to 50 °C (LT) and at high temperatures from 50 to 200 °C (HT). The pump power of the 980 nm laser was set at 100 mW. The real output power delivered to the TG05 glass sample (1 mm thick) attached to the commercial fibre bundle was approximately 30 mW when measured using a power meter equipped with an InGaAs detector. The use of low excitation power makes the proposed optical thermometer fibre probe suitable for use inside biological systems without the risk of damage to cells, proteins, and enzymes because 30 mW of excitation power does not cause sample heating. Details of the experimental setup and the apparatus used are presented in the Methods section and as Supplementary Material. The emission spectra for the LT and HT ranges are shown in Fig. 7. For the HT range, the emission intensity of the ^2^H_11/2_ level (525 nm) gradually increased with respect to the ^4^S_3/2_ level (548 nm) as a function of temperature owing to the increase in the electronic population of Er^3+^ at the ^2^H_11/2_ excited level at the expense of that of the ^4^S_3/2_ level by thermal excitation. The small energy gap between these two levels, approximately 799 cm^−1^ as estimated from the absorption spectrum, allows this thermally driven excitation. The relative electronic population of the thermally coupled ^2^H_11/2_ and ^4^S_3/2_ levels follows a Boltzmann-type population distribution given in Eq. (1).


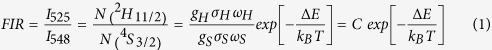


where *N, g, σ*, and *ω*, are the number of ions, the degeneracy of each level, the emission cross section, and the angular frequency of the fluorescence transitions from the ^2^H_11/2_ and ^4^S_3/2_ levels to the ^4^I_15/2_ level, respectively. Δ*E* is the energy gap between the ^2^H_11/2_ and ^4^S_3/2_ levels, *k*_*B*_ is the Boltzmann constant, and *T* the absolute temperature. The pre-exponential constant is given as 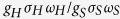.

An important parameter for evaluating the sensing ability of a particular active material for optical temperature measurements is the absolute thermal sensitivity, *S*, which is calculated as the rate at which the fluorescence intensity ratio changes with the temperature wherein the sensing element is applied^6^,^40^. The sensor thermal sensitivity can be compared by using the following figure of merit:


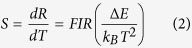


In order to compare sensitivity values obtained from different optical thermometry techniques and host material combinations, the relative thermal sensitivity, *S*_*R*_, is applied, which indicates the relative change of FIR (*R*) per degree of temperature, as defined in Eq. (3).


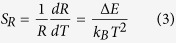


This parameter—which is expressed in units of percent change per degree Kelvin (% K^−1^)—was defined in 2007 in the context of temperature optical sensors^8^, and it has been commonly used as a figure of merit to compare different thermometers, independently of their nature^1^,^41^,^42^,^43^. Many authors employ absolute maximum sensitivity for comparison among different host materials; however, *S*_*R*_ has the advantage of being independent of the nature of the thermometer (i.e., mechanical, electrical, optical), thus allowing a quantitative comparison of different thermometry techniques. In this work, the absolute thermal sensitivity value was used for comparison in order to put this work in context within the literature, which largely uses *S*_*max*_ for comparison.

Table 1 presents the maximum values of the absolute thermal sensitivity (*S*_*max*_) at the maximum working temperature (*T*_*max*_), the relative thermal sensitivity (*S*_*R*_), the temperature range (Δ*T*), and the excitation wavelength (*λ*_*exc*_) for different glass host matrices doped with Er^3+^ or co-doped with Er^3+^/Yb^3+^. The largest value for absolute sensitivity is 9.1 × 10^−3^ K^−1^ at 150 °C (423 K), whereas the maximum sensitivity is 8.9 × 10^−3^ K^−1^ at 200 °C (473 K). It is worth noting that the sensitivity of the up-converted green emissions in the TG05 glass is one of the highest found in the literature when compared to different host materials, mainly those presented in Table 1, which are Er^3+^ and/or Er^3+^/Yb^3+^ doped. The *S*_*max*_ value for the TG05 sample is higher than those obtained for the Er^3+^:fluorotellurite glass of 5.4 × 10^−3^ K^−1^ at 274 °C (547 K)^28^, the Er^3+^/Yb^3+^ co-doped Al_2_O_3_ nanoparticles of 5.1 × 10^−3^ K^−1^ at 218 °C (491 K)^9^, the Er^3+^/Yb^3+^ -doped chalcogenide glass of 5.2 × 10^−3^ K^−1^ at 220 °C (493 K)^26^, and the Er^3+^/Yb^3+^ co-doped silicate glass of 3.3 × 10^−3^ K^−1^ at 23 °C (296 K)^44^, and it is much higher than that of 0.6 × 10^−3^ K^−1^ at 27 °C (300 K) reported for an Er^3+^ -doped fluorozirconate glass^45^. All results were obtained under excitation in the near-IR range.

In addition to the studies referred to in Table 1 it presents no systematic study of the optimum concentration ratio between Er^3+^ and Yb^3+^ to improve sensitivity of the optical thermometer. Most of them use co-doped systems containing low Er^3+^ and Yb^3+^ concentrations^9^,^26^,^27^,^46^,^47^ or single Er^3+^ -doped materials^10^,^28^,^45^,^48^. For example, Li *et al*.^44^ and Feng *et al*.^49^,^50^,^51^,^52^,^53^ used Er^3+^/Yb^3+^ co-doped silicate-based glass with high Yb^3+^ concentrations, which reduced the energy transfer efficiency owing to the high phonon energy of these glass hosts, lowering the thermal sensitivity of their materials. Comparing the low phonon energy of tellurite glass to that of silicate and other types of oxide glass, the TG05 sample showed better continuous transfer energy from the sensitizer (Yb^3+^) to the activator (Er^3+^) under excitation at 980 nm. The efficiently up-converted green radiation emitted carries temperature information. In our case, the Yb^3+^ concentration by volume of the TG05 glass was six times greater than the Er^3+^ concentration, keeping the concentration of sensitizer ions around the emitter ions very high. Thus, the relatively low concentration of Er_2_O_3_ (0.5 mol%) helped avoid reabsorption of the emitted photons. Lastly, our setup played a key role in improving the sensitivity during the response acquisition because of the use of a 1-mm-thick glass disk 10 mm in diameter in contact with the end of the fibre bundle. Comparison on thermal conductivity could not be done considering that none of the referred works provide information about the dimension and thermal conductivity of the glasses used for temperature sensing.

### Thermometer calibration

Instrument calibration is a very reliable procedure that relates the response of an instrument to any physical or chemical parameter (e.g. concentration of an analyte, temperature, etc.). Proper instrument calibration enables quantitative data analysis. In this study, we evaluated several metric parameters, which allowed us to evaluate the proposed optical thermometer performance and to make comparisons to other temperature sensors reported in the literature. Calibration functions based on the evaluation of the green FIR of the integrated emission band areas (A_1_/A_2_) ranging from 514.1 to 537.1 nm (A_1_) and from 537.1 to 566.4 nm (A_2_) as a function of temperature variation (°C) were established ranging from 5 to 50 °C and from 50 to 200 °C. For each temperature, the mean value of five replicate measurements was calculated. The calibration linearity was evaluated by the regression coefficient (*r*^2^), inspection of the residual plots, and the standard error of the regression (*s*_*y/x*_). The relative standard deviations (RSD) among measurements were found to be as low as 1% (*n* = 7), which translated to precisions of ±0.5 °C and ±1.1 °C in the ranges of 5–50 °C and 50–200 °C, respectively. The maximum temperature sensitivity of 8.9 × 10^−3^ K^−1^ was calculated at 150 °C, revealing the superior performance of our system among the many Er^3+^/Yb^3+^ co-doped materials for optical thermometry reported in the literature, as shown Table 1. The full calibration parameters and thermometer performance are summarised in Table 2.

Continuous incidence of the 980 nm radiation might have caused bias in the obtained data because of local heating, as previously discussed. Herein, we evaluated the stability of the A_1_/A_2_ signal under high- and continuous-radiation incidence with laser power at 400 mW (four times greater than that used for the temperature measurements). An intra-day measurement precision value better than 0.66% revealed that there was no significant interference in the A_1_/A_2_ signal, even after continuous exposure to the high-power input radiation.

### Thermometer accuracy

A water bath (Microquimica, Brazil) with a temperature range of 5 to 50 °C was used to evaluate the accuracy of the proposed temperature probe. For each temperature, the mean value of five replicate measurements was calculated and then compared with the temperature displayed in the water bath. A linear function between real and calculated temperature was established, which revealed excellent linearity (*r*^2^ > 0.99). Moreover, the paired t-test showed no statistically distinguishable difference at the 95% confidence level among the obtained data, showing excellent accuracy.

## Conclusions

A portable optical thermometer based on the UC green fluorescence intensity ratio of an Er^3+^/Yb^3+^ co-doped tellurite glass was developed by exploring the radiative transition from the thermally coupled ^2^H_11/2_ and ^4^S_3/2_ excited levels to the ^4^I_15/2_ ground state of Er^3+^. The designed system is useful for optical temperature sensing operating in two different temperature ranges according to the requirements: an LT range of 5–50 °C, with a precision of ±0.5 °C, for biological and biomedical applications, and an HT range of 50–200 °C, with a precision of ±1.1 °C, for applications such as non-contact sensors for temperature measurements, microelectronics temperature monitoring, and temperature measurement in harsh environments. An optimal concentration with a higher emission intensity was achieved by varying the Er^3+^ content. The TG05 tellurite glass sample (3 mol% Yb^3+^ and 0.5 mol% Er^3+^) presented many advantageous features owing to its highest green UC emission intensities, its low phonon energy, and high *S*_*max*_ value, and therefore it was shown to be more suitable for application as a temperature sensor. Its temperature sensitivity of 0.0089 K^−1^ at 200 °C (473 K) is one of the highest found in the literature for Er^3+^/Yb^3+^ co-doped tellurite glasses when 980 nm is used as the excitation source. The maximum relative thermal sensitivity was 0.53% K^−1^. We assume that the high Yb^3+^ concentration was mainly responsible for the high emission intensities owing to the large rate of energy transfer from Yb^3+^ to Er^3+^, making this material very sensitive to temperature variations.

## Methods

Co-doped tellurite glass samples with chemical compositions of (97−*x*)[70TeO_2_–15GeO_2_–5K_2_O–10Bi_2_O_3_]:*x*Er_2_O_3_/3Yb_2_O_3_ (mol%), where *x* = 0.1, 0.5, 1.0, and 1.5, were prepared by a conventional melt-quenching technique in a glove box purged with dry N_2_ (≤90 ppm water). The glass samples were denoted as TG01, TG05, TG10, and TG15, respectively. The commercially available raw materials (Prichem Tech, Alfa Aesar, Lumtech) used were TeO_2_, GeO_2_, K_2_CO_3_, Bi_2_O_3_, Er_2_O_3_, and Yb_2_O_3_ powders with 99.9% or higher purities. The starting materials were stoichiometrically weighed in order to obtain 10 g of glass bulk. The starting materials were thoroughly mixed in a translational mixer (model SpeedMixer) and then loaded into a gold crucible. The glass samples were melted at 700 °C for 0.4–1 h in a muffle furnace depending on the rare-earth concentration in order to ensure complete elimination of adsorbed gases and good homogenisation. The glass melts were cast into a preheated stainless steel mould, annealed at 20 °C below *T*_*g*_ for 2 h, and then slowly cooled to room temperature to minimise residual internal stress. Optical-quality polished glass samples were used for optical characterisation. Ground and sieved glass samples (0.063 mm > grain size > 0.045 mm) were used for the preliminary photoluminescence measurements to allow for a reliable comparison of the luminescence intensities. Working as a sensor element, an optically polished TG05 glass bulk sample was used in the proposed portable optical thermometer.

The characteristic temperatures (*T*_*g*_ for glass transition and *T*_*x*_ for the onset of crystallisation) were obtained for the glass samples using differential scanning calorimetry (DSC) at 200 to 600 °C under N_2_ at a heating rate of 10 °C/min using a TA Instrument Model 2910 calorimeter, with a maximum error of ±2 °C for both *T*_*g*_ and *T*_*x*_. The thermal stability against crystallisation was evaluated from the stability parameter, Δ*T = T*_*x*_ – *T*_*g*_. UV–visible (vis)–near infrared (NIR) absorption spectra were recorded on polished TG glass samples 1 mm thick using a Cary 500 (Varian) double-beam spectrometer. The linear refractive indexes of the samples were measured by using the prism coupling technique (Metricon 2010) at three different wavelengths, i.e., 532, 632.8, and 1550 nm. At first, the green UC emissions of the glass samples were evaluated using a Horiba Jobin Yvon fluorimeter equipped with a photomultiplier tube (PMT) sensitive from 250 to 850 nm. A diode laser operating at 980 nm coupled with a standard multimode fibre (50 μm core diameter) was used to excite the powdered glass samples by focusing on the sample surface through a lens with a 13-mm focal length. These measurements were performed at room temperature (~28 °C) and corrected by the instrumental response. For a reliable comparison between the luminescence spectra of the different samples, the glass samples were ground and sieved to obtain similar grain size distributions from 0.063 to 0.045 mm for all samples. The sample holder used for luminescence measurements had an elliptical shape of 10 mm × 5 mm and a 1 mm depth; it was positioned at an angle of 30° relative to the detector. In this way, by using the same powder glass volume and grain-size distribution, we expected similar scattering of the excitation light for all samples, allowing for a reliable comparison of the emission intensities among the different glass samples^38^.

In the second step, the temperature setup used to calibrate the optical thermometer included a drilled block of aluminium (60 × 45 × 2 mm), a drilled block of Teflon^®^ (60 × 45 × 10 mm), a polyimide foil heater (specs: 10 W/in^2^, Omegal), and a Peltier element (specs: 5 A, 15.4 V, 44.5 W, dimensions: 40 × 40 mm), with the hot side pasted on a commercial CPU cooler (Cooler Master). A voltage/current controller (MPL-1303M, Minipa) was coupled to handle the thermal elements. This setup enabled handling of temperatures from 5 to 200 °C. The aluminium block was positioned at the bottom, enabling high thermal conductivity among the thermal elements. A rubber layer (60 × 45 × 5 mm) was inserted between the aluminium and Teflon^®^ blocks. A portable calibrated digital thermocouple (HM-2010, Hikari) was positioned near the measurement point as a temperature standard controller.

**Figure 8:**
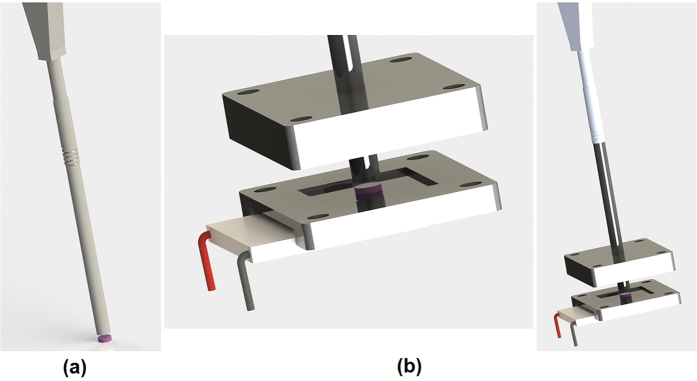
(**a**) Three-dimensional rendered model of the temperature probe and (**b**) the heater/cooler used to calibrate the sensor. A Peltier element and a polyimide foil heater were positioned at the bottom.

Our proposed temperature probe is shown in the Supplementary Material. It was composed of a commercial fibre bundle (model RP-24, Thorlabs) attached to the TG05 glass bulk sample, which was 1 mm thick with a diameter of 10 mm [Fig. 8(a)]. During the calibration step, the probe set was inserted into the heater/cooler through a dimensioned hole located at the top of the Teflon^®^ block [Fig. 8(b)], and the temperature was adjusted using the voltage/current controller. On the other side of the fibre bundle, one channel of the bundle was placed face-to-face with the standard multimode fibre coupled with a diode laser emitting at 980 nm with 100 mW. The other bundle channel was coupled to portable spectrometers, Ocean Optics model HR2000 + ES (for the LT range) and Ocean Optics model USB4000-FL (for the HT range). The spectrometers were sensitive from 190 to 1100 nm, with an optical resolution of 0.9 nm a full width at half maximum of 10 nm, and integration times of 850 and 1000 ms for LT and HT, respectively.

The UC emission spectra were recorded in the wavelength range from 400 to 600 nm, with an integration time of 100 ms at different temperatures from 5 to 200 °C. The Spectra Suite software package (Ocean Optics, USA) was used for data acquisition.

## Additional Information

**How to cite this article:** Manzani, D. *et al*. A portable luminescent thermometer based on green up-conversion emission of Er^3^^+^/Yb^3^^+^co-doped tellurite glass. *Sci. Rep.* **7**, 41596; doi: 10.1038/srep41596 (2017).

**Publisher's note:** Springer Nature remains neutral with regard to jurisdictional claims in published maps and institutional affiliations.

## Figures and Tables

**Table 1:** Values of the maximum sensitivity and relative sensitivity of different Er^3+^ and Er^3+^/Yb^3+^ co-doped materials using the FIR method to obtain the green emissions of the Er^3+^.

Doped materials	*S*_*max*_(10^−3^ K^−1^)	*S*_*R*_^§^ (% K^−1^)	*T*_*max*_ (°C)	Δ*T* (°C)	*λ*_*exc*_(nm)	Ref.
Er^3+^/Yb^3+^:tellurite glass	8.9	0.53	200	5–200	980	This work
Er^3+^:fluorotellurite glass	5.4	0.35	267	27–267	800	28
Er^3+^/Yb^3+^:chalcogenide glass	5.2	0.38	220	20–220	1060	26
Er^3+^/Yb^3+^:Al_2_O_3_ nanoparticle	5.1	0.40	218	22–700	978	9
Er^3+^/Yb^3+^:germanate tellurite glass	3.6	0.39	220	20–220	976	46
Er^3+^/Yb^3+^:silicate glass	3.3	0.63	23	23–450	978	44
Er^3+^:fluoroindate glass	2.8	0.55	152	−150–152	980	48
Er^3+^:oxyfluoride glass	2.7	0.41	240	−23–177	980	47
Er^3+^:PbO-Ga_2_O_3_-SiO_2_ glass	2.6	0.24	317	23–377	980	10
Er^3+^/Yb^3+^:TeO_2_–WO_3_ glass	2.6	0.20	417	27–417	980	27
Er^3+^/Yb^3+^:fluorophosphate glass	1.5	0.71	6	−196–227	980	49
Er^3+^:fluorozirconate glass	0.6	0.58	27	−123–577	976	45

The temperature range, excitation wavelength, and temperature for the maximum sensitivity values are also included. ^**§**^*S*_*R*_ values were extracted by the authors from the references data (WebPlotDigitizer).

**Table 2:** Full calibration parameters of the thermometer in its dedicated temperature ranges.

Parameter	Working temperatures	
	LT range 5-50°C	HT range 50-200°C
Slope (*a*)	0.00219 (±0.0000547)	0.00761 (±0.000237)
Intercept (*b*)	0.310 (±0.00154)	0.381 (±0.0291)
*r*^2^	0.9961	0.9919
Regression SD (*s*_*y/x*_)	0.002193	0.0358
Precision (RSD)	0.54	0.95
Precision (±°C)	0.5	1.1
